# Spectral Properties of Marennine-like Pigments Reveal Minor Differences Between Blue *Haslea* Species and Strains †

**DOI:** 10.3390/molecules29225248

**Published:** 2024-11-06

**Authors:** Amina Latigui, Boris Jacquette, Jens Dittmer, Jean-François Bardeau, Edouard Boivin, Lucie Beaulieu, Pamela Pasetto, Jean-Luc Mouget

**Affiliations:** 1 Institut des Molécules et Matériaux du Mans, Le Mans Université, Avenue Olivier Messiaen, 72085 Le Mans, France; amina.latigui@univ-lemans.fr (A.L.); boris.jacquette@univ-lemans.fr (B.J.); jens.dittmer@univ-lemans.fr (J.D.); jean-francois.bardeau@cnrs.fr (J.-F.B.); edouard.boivin@univ-lemans.fr (E.B.); 2 Laboratoire Biologie des Organismes, Santé, Environnement, Le Mans Université, Avenue Olivier Messiaen, 72085 Le Mans, France; 3 Institute of Nutrition and Functional Foods (INAF), Université Laval, Québec, QC G1V 0A6, Canada; lucie.beaulieu@fsaa.ulaval.ca; 4 Department of Food Science, Faculty of Agricultural and Food Sciences, Université Laval, Québec, QC G1V 0A6, Canada; 5 Québec-Océan, Université Laval, Québec, QC G1V 0A6, Canada

**Keywords:** diatoms, *Haslea*, marennine, marennine-like pigments, spectral properties

## Abstract

Marennine is the specific bluish pigment produced by the marine diatom *Haslea ostrearia* Gaillon (Simonsen), responsible for the greening of oysters in France’s Atlantic coast. For decades, *H. ostrearia* was considered the only blue diatom and described as such. However, new blue *Haslea* species have been described recently, among which *Haslea karadagensis* Davidovich, Gastineau, and Mouget (Black Sea, Crimea, Ukraine); *Haslea provincialis* Gastineau, Hansen, and Mouget (Mediterranean Sea, southern France)*; Haslea silbo* Gastineau, Hansen, and Mouget (West Atlantic Ocean, USA); and one not characterized yet, *Haslea* sp. nov., isolated in Tenerife (Spain). These species produce marennine-like pigments, for which little information is available yet. The present work aims at studying spectral characteristics of these pigments by UV–visible spectrometry, Raman spectrometry, infrared spectrometry, nuclear magnetic resonance, energy-dispersive X-ray spectroscopy, and cyclic voltammetry, and comparing them to those of marennine produced by *H. ostrearia* strains originating from the north Atlantic Ocean (western France and Macaronesia), and north Pacific Ocean (southwestern USA). Results show that marennine produced by *H. ostrearia* strains and marennine-like pigments produced by *H. provincialis*, *H. silbo*, and *Haslea* sp. nov. are quite similar regarding their polysaccharide skeleton, and absorption in the UV–visible, infrared, and Raman regions. The most different marennine-like pigment is produced by *H. karadagensis,* but all *Haslea* blue pigments studied so far belong to the same family of organic molecules.

## 1. Introduction

Marennine, a blue-green pigment, is synthesized by the marine diatom *Haslea ostrearia* [1]. For many decades, *H. ostrearia* was considered the only diatom species capable of synthesizing marennine. Other species of blue *Haslea* that produce similar blue pigments, known as marennine-like pigments [2], have recently been identified. The second blue *Haslea* species ever described, *Haslea karadagensis*, was collected in the Black Sea in Crimea (Ukraine) [3]. Then, *Haslea provincialis* was identified in the western Mediterranean Sea [4], *Haslea nusantara* in the Java Sea (Indonesia) [5], and *Haslea silbo* at Gran Canaria of the Canary Islands (Spain) [6]. *Haslea* blue pigments are responsible for the natural greening of oysters and other marine invertebrates’ gills [7,8]. The “fines de claire vertes” oysters are considered to have superior organoleptic qualities due to the presence of marennine [9]. Moreover, these so-called “green oysters”, because of their relative rarity, are more expensive than their non-colored counterparts [10]. In addition to coloring gills, marennine-like pigments also have antibacterial, antiviral, antioxidant, and allelopathic properties [8,11,12].

Over two centuries, different hypotheses were proposed regarding the chemical nature of marennine [13]. Some authors linked marennine to a carotenoid-like compound [14], or an anthocyanin-like compound [15]. The first in-depth investigations refuted all of them and suggested a complex molecule with an aromatic chromophore [16] bound to a polysaccharidic skeleton [2,17]. Furthermore, Pouvreau et al. [16] evidenced the existence of two distinguishable forms of marennine, extracellular (EMn) and intracellular (IMn), but little is known about differences with other marennine-like pigments. Moreover, the isolation, purification and precise identification of the chemical structure of marennine have long been challenges. What has been misleading for a long time is the search for a single organic molecule as responsible for the blue color, while ongoing works suggest that the chromophore is possibly enveloped by oligosaccharides, thus the difficulty in defining its nature. Researchers have used nuclear magnetic resonance (NMR) and mass spectrometry to attempt to characterize marennine [16,17], but its exact structure remains to be determined because of the complexity resulting from the binding of different chemical entities. Recently, some information came from the study of marennine electrochemical behavior [18], and it was confirmed that the redox properties observed were due to the chromophore and not to the entire complex. The chromophore has also been found to be resistant to the acidic and basic treatments used for its isolation [19]. Nevertheless, during the purification and recovery of marennine, it demonstrates a valuable stability by keeping a constant color, which changes only depending on the pH and redox environments [18]. These characteristics will facilitate the incorporation of marennine in formulations for different applications, offering a sustainable production of natural pigments, a point of great interest for the emergence of bioeconomy. Indeed, natural products have less impact on the environment and on consumers’ health [20]. Currently, only phycocyanin has been approved by the U.S. Food and Drug Administration (FDA) as a blue dye. Unfortunately, phycocyanin is sensitive to temperature, light, and pH; therefore, marennine could be a promising substitute, as its blue color remains stable after heating at 100 °C in acidic and basic pH. In view of scaling up the production of pigments for industrial applications, a few attempts have been made to enhance diatom cultures, for instance, using indoor culture by the Société de Production de Microalgues (Soproma, Bouin, France). They exploited a patent process that describes the culture conditions (composition of the medium and irradiation [21]) to grow *H. ostrearia* using underground seawater, with the objective to green oysters on demand all year long. The economic viability of the culture process has not been demonstrated, and ongoing works along this line explore the feasibility to grow the microalgae in photobioreactors [22] and to concentrate the pigments out of the supernatants using precipitation and purification [19], or ultrafiltration techniques with immersed membranes [22,23].

Although it has been demonstrated that blue *Haslea* pigments exert various biological activities and are of great interest in several industries that exploit blue dyes, in particular the textile and cosmetic sectors, marennine-like pigments remain underutilized mostly due to incomplete data on their properties and limited availability. Even less is known about the other, recently discovered blue *Haslea* species and their marennine-like pigments. To fill this gap, the present work aims at characterizing extracellular marennine-like pigments produced by different species of blue *Haslea* diatoms, as well as strains of *H. ostrearia* of different geographic origin. The available strains examined at the time of this work were three strains of *H. ostrearia* sampled in the Bay of Bourgneuf (BB) on the Atlantic coast of France, in the San Diego Bay on the Pacific coast of California (SD), and in Puerto de Mogán (PM) in Gran Canaria (Canary Islands, Spain); *H. provincialis* collected in the Bay of Calvi (Corsica, France); *H. silbo* collected at Morehead City Channel in North Carolina (USA); and the not yet characterized *Haslea* sp. nov*.,* sampled in Puerto de los Abrigos in Tenerife (Canary Islands, Spain). After isolation, the microalgae were grown in laboratory and culture supernatants were submitted to the procedure of purification described in the patent FR2019/052933. The obtained pigments were characterized by NMR to compare the proton spectra to the one of marennine produced by *H. ostrearia* [17], which had allowed for the identification of different sugars and types of glycosidic bonds in the oligosaccharides. Raman and infrared signatures were compared to deduce differences or similarities, as Raman spectra of intracellular and extracellular forms of marennine, as well as of green oyster gills, have been already recorded for *H. ostrearia* [13]. The UV–visible spectra were carried out at different pH values to observe the shift of the blue-green color, and cyclic voltammetry was carried out to monitor the change of the oxidation state in relation to the change blue-yellow color. Energy-dispersive X-ray spectroscopy analysis was also carried out on the dry powders to determine the atomic composition. The data collected so far showed minor differences between blue *Haslea* species and strains in terms of sugar composition and chromophore characteristics, thus demonstrating they belong to a same family of pigments.

## 2. Results and Discussion

### 2.1. UV–Visible Spectrometry

**Figure 1:**
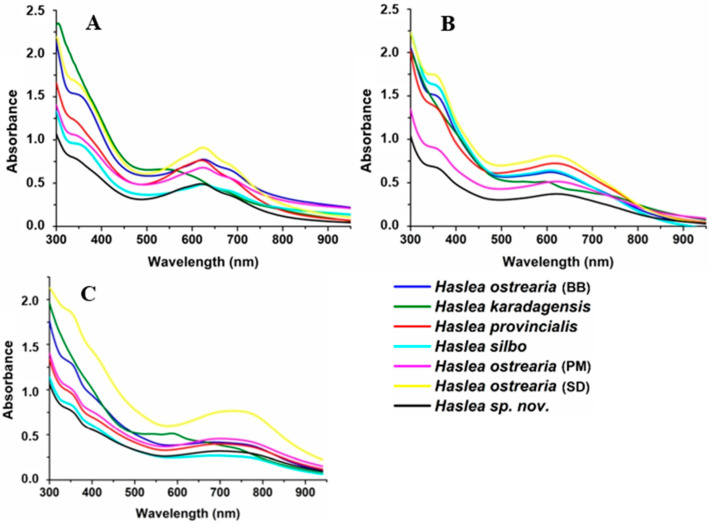
Comparison of UV–visible absorbance of marennine-like pigment solutions at pH 1 (**A**), 7 (**B**), and 10 (**C**) of different *Haslea* species (for all strains concentration was 2 mg/L, in 0.2 M phosphate buffer).

Using light microscopy, blue *Haslea* cells are easy to spot in periphyton samples mounted in slides due to the blue color at their apices (Figure S7). When blue *Haslea* cells grow and bloom in closed environments, excreted extracellular marennine or marennine-like pigments make seawater or culture supernatants blue-green, depending on the pH, so it is obvious to first characterize the new species by their absorption in the UV–visible region of the electromagnetic spectrum. As marennine from *H. ostrearia* changes color with the pH of the medium, solutions were prepared in different phosphate buffers in the range of pH 1–10. Not knowing the chemical formula of the molecules, hence their molecular weight, the same mass of purified pigment was weighed and dissolved in the same volume of solvent. Spectra were taken in the spectral range 300–950 nm (Figure 1, comparison between pH 1 (A), 7 (B), and 10 (C) for all species; complete characterization of each species in the range of pH 1–10 is reported in Figure S1). All pigments absorbed in the UV region, where a peak was visible around 350 nm, and it was not influenced by pH variation. The presence of this band could be due to an aromatic ring or a conjugated system in the chromophore, as sugars or lipids do not usually absorb in the UV region. In the visible region, at acidic pH, the maximum absorbance wavelength was around 625 nm, with a shoulder at 590 nm and another at 700 nm for all species except *H. karadagensis*, which had a large band centered at 537 nm. At neutral pH, the maximum absorption wavelength remained around 625 nm but the absorbance of the two shoulders diminished for all species, except *H. karadagensis*, for which the spectrum changed and two bands appeared, centered at 600 nm and 750 nm. At basic pH, the absorbance was quite a large band; however, the wavelength of the maximum absorbance shifted toward 800 nm; again, the exception was *H. karadagensis,* for which the spectrum was similar to the one at pH 7. The chromophore responsible for small absorption changes its chemical structure with the pH, suggesting that a protonated positively charged form could be in equilibrium with a deprotonated neutral one, and an anionic one at basic pH. The different delocalization of the electrons in each form could explain the different energy of the electronic states, hence the changes in color. As the spectra of the different strains did not exactly overlay, this could mean that there are minor differences in pigment between species, such as the presence of an additional group on an aromatic ring that could slightly influence the conjugation systems. For *H. karadagensis* the difference in the chemical structure must be more relevant, as its behavior differs significantly from the others.

### 2.2. Study of Redox Behavior

The relation between redox state and color was previously studied for marennine from *H. ostrearia* (BB) [18,19], therefore, the study was repeated for the new pigments. It was found that the chromophore was responsible for the redox reaction [24], as a change in color from blue to yellow (hypsochromic shift in the maximum wavelength towards 400–500 nm) occurred when reducing chemicals, such as sodium dithionite, were added in solution. It appeared that the blue color in acidic pH is associated to an oxidized form of the chromophore, while the yellow one corresponds to a reduced form, which is unstable in presence of oxygen, giving an intermediate deep blue state before going back to the native one. From these experiments, it emerged that there are at least two redox couples; an estimation of the standard redox potentials could be made, and visible spectra were carried out to associate colors to the electrochemical potential.

**Figure 2:**
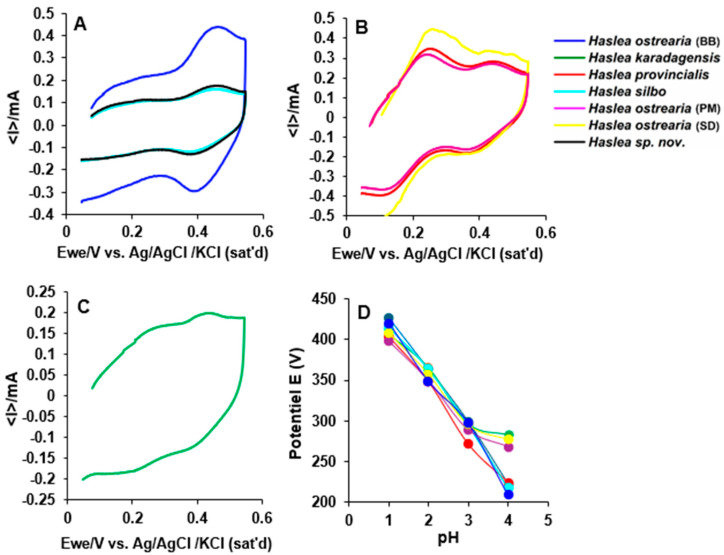
Cyclic voltammograms at 100 mV/s of marennine and marennine-like pigment solutions (1 mg/mL), using a glassy carbon working electrode and platinum. At pH 1: (**A**) *H. ostrearia* (BB), *H. silbo*, and *Haslea* sp. nov.; (**B**) *H. provincialis*, *H. ostrearia* (SD), and *H. ostrearia* (PM); (**C**) *H. karadagensis.* (**D**) potential E (V) vs. various pH in the range of 1–4.

For the new pigments, voltammograms could be obtained only in acidic solutions for pH ≤ 4 (Figure 2 and Figure S2). In Figure S2 all voltammograms for each species in the range of pH 1–4 are displayed). At pH 1, the species could be classified into two groups displaying either one (at 420 ± 5 mV vs. Ag/AgCl), as observed for *H. ostrearia* (BB), *H. silbo*, and *Haslea* sp. nov. or two redox contributions (405 ± 5 mV vs. Ag/AgCl and another at lower potential), as observed for *H. provincialis*, *H. ostrearia* (SD), *H. ostrearia* (PM), and *H. karadagensis.* However, the low potential peak observed for this latter species is significantly different from the others (215 ± 5 mV against 175 ± 5 mV vs. Ag/AgCl for *H. provincialis*, *H. ostrearia* (SD), and *H. ostrearia* (PM)). Hence, at least four different redox-active centers were identified, with potential of 420 ± 5 mV, 405 ± 5 mV, 175 ± 5 mV, and 215 ± 5 mV vs. Ag/AgCl at pH = 1, respectively. As the redox potential decreased with the pH, this means that H^+^ is a product of the associated oxidation reactions, according to the Nernst equation. However, a slope corresponding to the production of 1 H^+^/e^−^ (*ca.*—60 mV per unit of pH) was only observed for *H. ostrearia* (*BB*), *H. silbo*, *Haslea* sp. nov., and *H. provincialis,* while ca. —40/−45 mV/pH (i.e., between 2/3 H^+^/e^−^ and ¾ H^+^/e^−^) was measured for *H. karadagensis*, *H. ostrearia* (PM), and *H. ostrearia* (SD). Considering both redox potential and their evolution with the pH, at least six different redox-active centers could be identified. Moreover, as the pH increased, the intensity of the current diminished until no significant faradaic contribution could be detected above pH 4. As H^+^ is a product of the reaction, this indicates that only the acidic form of the species are redox-active and that their pK_A_ are approximately 4–5. Furthermore, for the species displaying two redox peaks, redox reactions involving an intermediate state can be excluded since the intensity ratios between two consecutive contributions do not seem correlated and hence possess at least two distinct redox centers.

The changes in the visible spectrum observed by changing both the pH and the redox state mean that a rearrangement takes place in the chromophore in a protonated form. The peak in the UV region is less influenced, as the maximum wavelengths remained at 350 nm, with a slight decrease in absorbance [18]. Under highly acidic conditions, the pigments of *H. provincialis*, *H. ostrearia* (PM), and *H. ostrearia* (SD) showed high electronic exchange capacity, unlike *H. ostrearia* (BB), possibly due to the structural specificity of these pigments. Furthermore, the curves of *H. karadagensis*, *H. silbo*, and *Haslea* sp. nov. exhibited less pronounced redox activity, probably due to fewer reversible redox mechanisms due to distinct molecular pigment interactions that influence their protonation. The diversity of responses observed highlights the influence of the chemical structure of the pigments and the intra- and interspecific variation between the marennine-like pigments produced by these blue *Haslea*. The ability of some strains to maintain their redox activity even under extreme conditions may confer survival advantages and indicates potential for biotechnological applications such as cosmetics, anticancer drugs, or the food industry, where pigment stability is essential.

### 2.3. Infrared Analyses

**Figure 3:**
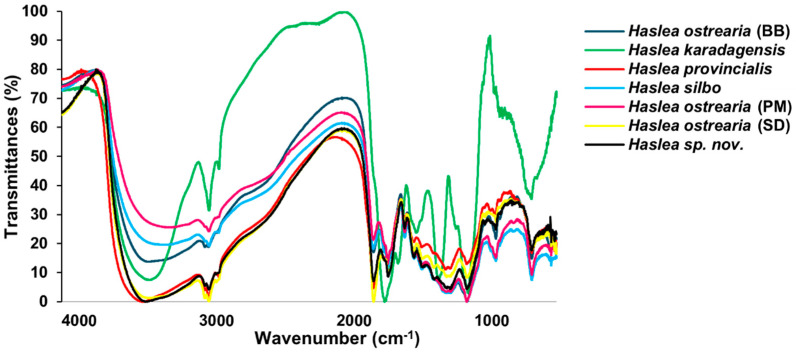
FT-IR spectra obtained from different blue *Haslea* pigments.

Infrared spectroscopy can be used to characterize pigments in terms of information about their molecular structure. Even if the technique cannot give detailed information about a molecular formula, the fingerprint region is so unique that it can be overlaid between species and show differences or similarities very quickly. Samples from all *Haslea* strains showed a broad and intense peak around 3500 cm^−1^ (Figure 3 and Figure S3, Table 1) that is typically associated with stretching of hydroxyl O-H bonds [25,26], which could be the ones on the oligosaccharides. Intense and thin peaks were also observed between 3000 and 2800 cm^−1^, corresponding to the stretching of aliphatic C-H bonds in -CH, -CH_2_, and -CH_3_ moieties [27,28]. At least two peaks can be attributed to the stretching of carbonyl bonds, one around 1700 cm^−1^ and the second around 1600 cm^−1^ [29]. The first could belong to ester groups and the second to acidic ones, as the signals for these two groups generally appear in this wavenumber order [30]. These bands should be related to the peaks at 1100 cm^−1^, which could belong to the stretching of the C-O bond in esters and carboxylic acids, but also to C-O in glycosidic bonds. In the region 1690–1655 cm^−1^, peaks for quinones, which have both carbonyl groups in the same ring, can also be found. The spectral absorption peaks at 1528 and 1487 cm^−1^ suggest the presence of C=C bonds. Distinct peaks observed in the region of 1200–900 cm^−1^ could be related to the polysaccharides [31].

The spectra for six pigments are similar, almost superimposable, the exception being again *H. karadagensis*, which has a different signature in the fingerprint region (especially between 1500 and 1000 cm^−1^), suggesting the presence of carbonyl groups in different configurations.

### 2.4. Raman Spectroscopy

**Figure 4:**
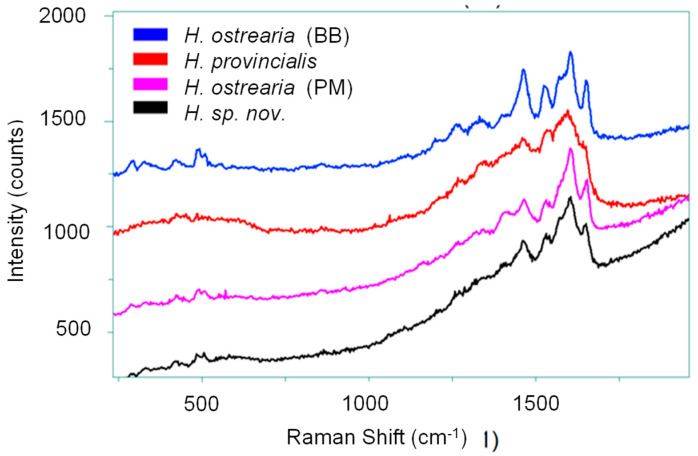
Raman spectra of marennine-like pigments of different strains and species of blue *Haslea*.

Raman spectroscopy is a well-known non-destructive chemical analysis, complementary to infrared spectrometry, used in different disciplines, particularly for the analysis of dyes and pigments. It has played a crucial role in the preliminary exploration, both in vivo and in vitro, of marennine-like pigments produced by different species of blue *Haslea*, thereby facilitating their differentiation [3,4]. The Raman spectra obtained for the pigments of *H. ostrearia* (BB), *H. provincialis*, *H. ostrearia* (PM), and *Haslea* sp. nov. show similar characteristics (Table 2), of a nature that can be ascribed to various chemical bonds within the pigment. The peaks around 1462 and 1527 cm^−1^ are probably associated with the C=C vibrations of the conjugated bonds of the chromophores, as shown in Figure 4 and Figure S4. The 1647, 1650, and 1651 cm^−1^ peaks of *H. ostrearia* (PM), *H. ostrearia* (BB), and *Haslea* sp. nov. suggest a C=O bond vibration, often found in carotenoids and other organic pigments. These bond vibrations have also been observed in *H. karadagensis* and *H. silbo* [3,6].

### 2.5. Nuclear Magnetic Resonance

**Figure 5:**
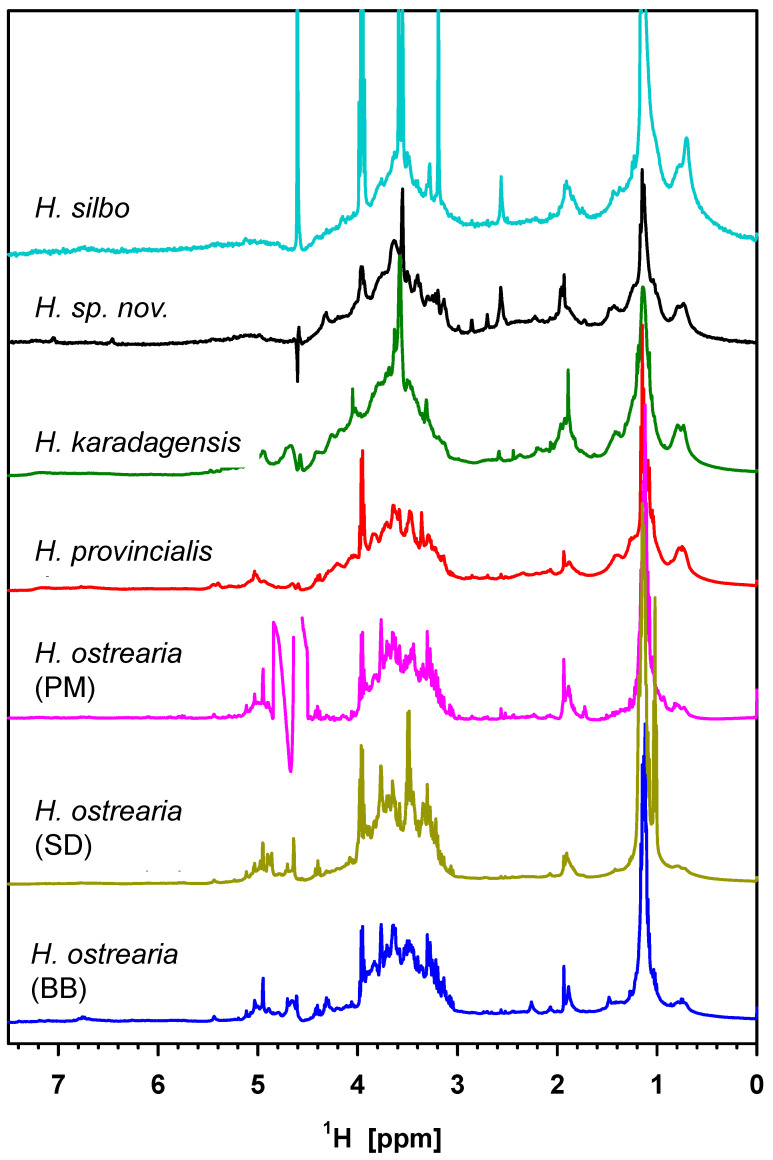
^1^H-NMR spectra of purified marennine-like pigments in D_2_O. The residual water signal and three high-impurity signals of *H. silbo* are cleaved in the figure. In the spectrum of *H. ostrearia* (PM), the region beyond 4.8 ppm is compromised by insufficient water suppression.

^1^H NMR is—in principle—a quantitative method to assess the organic composition of a sample. The signals, however, broaden as a function of the size of a molecule so that small signals appear overemphasized. The NMR spectra of purified marennine-like pigments dissolved in D_2_O (Figure 5) must be interpreted in this context. Most of the prominent sharp signals stem from residual impurities or fragments of degraded molecules. The actual signals of interest are overlapping to groups due to their number and their line widths. Overall, the spectra of all samples show the same constituents (although this is less apparent in *H. silbo* due to dominant impurity signals). The main constituents are a signal group between 3.0 and 4.5 ppm, representing sugar ring protons of polysaccharides, confirmed by the smaller anomeric signals between 4.6 and 5.2 ppm [17,35]. The high signal at about 1.1 ppm is composed of the methyl signals of sugars containing such groups as rhamnose and fucose (vide infra) and CH_2_ chains of other origin, together with their methyl group at 0.8 ppm [17]. The signals around 2 ppm are reproduced and are partly assigned to N-acetylgluco- or galactosamine. Despite the overall similarity, one can discriminate the sugar ring group of the three *H. ostrearia* strains from the other species. Differences can be due to monosaccharide composition but also due to typical glycosidic bonds, as the neighbor type and position have a strong impact on the chemical shift.

On the other hand, the signal positions of monosaccharides are well defined. They only might vary from reference to reference, but the relative distances are well reproducible.

The pigment of *H. provincialis* was hydrolyzed as described in [17] and ^13^C spectra were acquired, which generally provide a spectral resolution sufficient to unambiguously identify and quantify the sugar composition.

**Figure 6:**
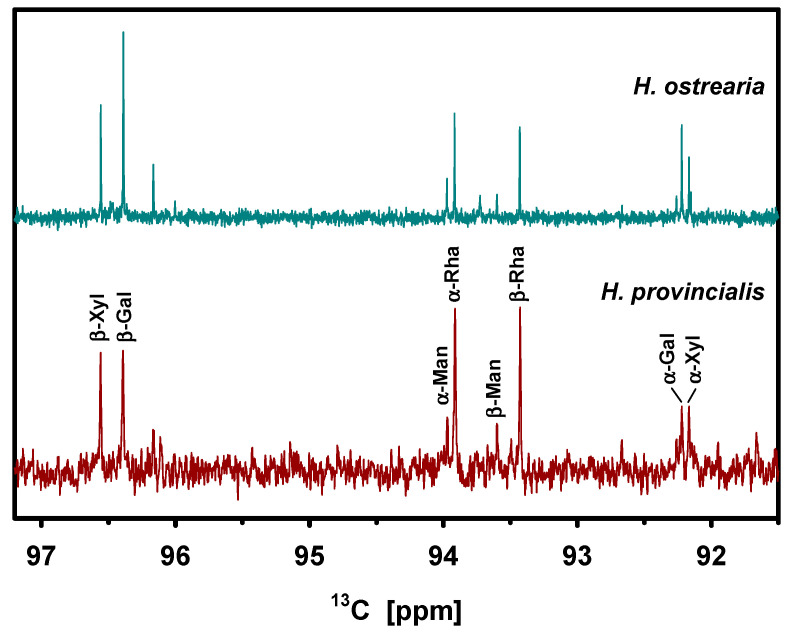
Anomeric region of the ^13^C DEPT spectrum of *H. provincialis* hydrolyzed pigment compared to that of *H. ostrearia* (BB) [17].

Figure 6 shows as an excerpt the region with the signals from the anomeric carbons. It shows high similarity with the spectrum of *H. provincialis*, except for that the highest signals stem from rhamnose rather than *β*-galactose. Note that the existence of both *α*- and *β*-conformers are due to hydrolysis and does not reflect the ratio in the polymer. The relative signal intensities match well the natural ratio. Table 3 lists the percentages of the identifiable monomers as determined by integration and addition. The signal of fucose hardly exceeds the noise (*β*-Fuc at 96.2 ppm), but the methyl signals of *α*- and *β*-fucose coincide at 15.4 ppm and allow for quantification.

The agreement of *H. provincialis* and *H. ostrearia* is also apparent numerically, except for the interchange of rhamnose and galactose. This reassures again that the polysaccharide isolated together with the chromophore is really related to the latter rather being mainly some more or less arbitrary extracellular polysaccharide.

Confirming many previous observations, it is still surprising that there are no reproducible signals in the spectra that can be attributed to a chromophore, such as aromatic (7–7.5 ppm) or other sp^2^ hydrogens such as conjugated double bonds, which would all resonate beyond 5.5 ppm ^1^H.

### 2.6. Energy-Dispersive X-Ray Spectroscopy

**Figure 7:**
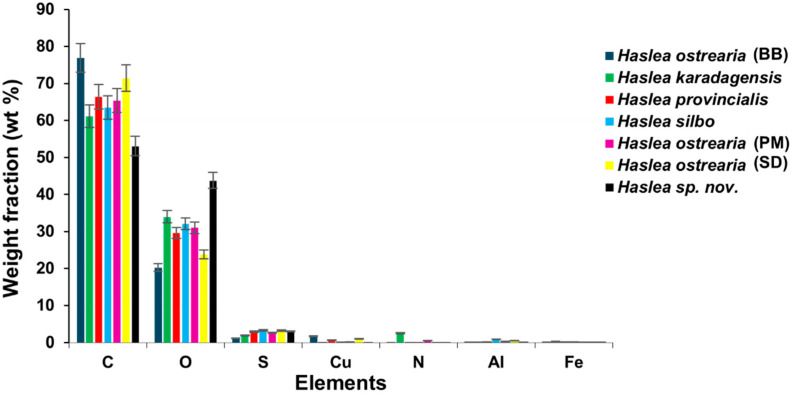
Elemental composition of blue *Haslea* pigments by energy-dispersive spectroscopy.

Semi-quantitative analyses of the elemental composition of the pigments from various *Haslea* strains as powders were conducted using energy-dispersive X-ray spectroscopy (EDX) coupled with scanning electron microscopy (SEM). Their atomic composition was identified by the detection and quantification of various elements, except hydrogen and helium. The results revealed that carbon (C) and oxygen (O) dominate the elemental composition with respective averages of 62 ± 2% and 30 ± 2% (Figure 7, Figures S5 and S6, Table 1). The proportions of nitrogen (N) and sulfur (S) were lower, averaging around 2%. Concentrations of trace elements such as sodium, aluminum, iron, and copper were less than 1%. The presence of sugars revealed by NMR analyses explains the predominant presence of carbon and oxygen. The variations observed in the elemental composition of different marennine-like pigments could reflect differences in species metabolism, physiological state, or adaptation to the environment. Indeed, the culture conditions ensuring pigment production were initially defined for *H. ostrearia* (BB) and were the same for all blue *Haslea* strains. Given the different geographic origin of other blue *Haslea* strains, these culture conditions are possibly not optimal for all of them. The presence of nitrogen is relatively modest, as observed by Pouvreau, who discarded the hypothesis of a protein nature for marennine. Nitrogen is probably on glucosamine or galactosamine sugars, as revealed by NMR analyses [17,35]. The traces of copper are insufficient to support the hypothesis of a metal complex as the primary source of coloring. The presence of sulfur is probably due to the sulfate groups on the polysaccharidic chains, as observed from the electrophoresis carried out on the oligosaccharides separated from the chromophore of *H. ostrearia* (BB) (unpublished results).

### 2.7. Different Species and a Unique or a Family of Pigments?

Until now, after bio-prospection in the sea and isolation of microalgae with blue tips in the samples, three criteria were retained to assess if the isolated diatoms belonged to an already known *Haslea* species: the morphology of the frustule; some DNA molecular markers; sexual reproduction. The shape and features of the silica frustules were observed using scanning electron microscopy and striae density compared; DNA was extracted and sequences were compared; every time possible, clonal cultures of newly discovered blue *Haslea* were crossed with every known species (both sexual types) already in culture in the lab. By doing so, new species for science have been described, and phylogenetic trees reveal their evolutionary proximity [22]. In complement to these criteria, and despite our limited knowledge about the chemical structure of marennine, UV–visible and usually Raman spectra of marennine-like pigments were recorded of the newly described species of blue *Haslea*, *H. karadagensis*, *H. provincialis*, *H. nusantara*, and *H. silbo*. From these spectra, it was only possible to infer a significant difference between the pigment produced by *H. karadagensis* and the pigments produced by all other species. Using a wide combination of spectral characterization methods, our results confirm this feature, but evidence only little differences between other marennine-like pigments, which possibly reflect the absence of major structural specificities, in particular at the chromophore level.

If the chromophore is an organic molecule, the slight differences observed in the infrared spectra could come from the glucidic moiety, for instance, from signals belonging to simple and double carbon–oxygen bond stretching (region around 1100 cm^−1^ and 1600 cm^−1^, respectively), indicating the presence of uronic acids or carbonyl groups of from esters or ethers. The protons from sugars also provide the major signals observed in the nuclear magnetic resonance spectra, so that if the chromophore is a small aromatic molecule with a high extinction coefficient, its concentration in the deuterated solution would be so small as to be nearly undetectable, or its signals could be decreased by the presence of a paramagnetic element.

UV–visible spectra reflect the distribution of the electronic levels, and the differences could come from different substituents on an aromatic ring or from different isomers, such as quinone groups in ortho or para positions. Being the color sensitive to pH, a protonation–deprotonation equilibrium also takes place, possibly due to the switching between two colored isomeric forms. The voltammetry data come from the chromophore; they reveal the existence of several redox centers, but again, these are only slight changes that could be due to the different position of the same substituent or a group; they are not intense enough to distinguish between two completely different families of organic molecules.

Regarding possible environmental cues explaining these small differences, the main parameter that distinguishes the Black Sea from the other seas and oceans is the salinity, which is ca. 50%, but it seems difficult to explain how this could be correlated to a different molecular structure. Average sea temperature does not seem to affect the color either, as the similarity between UV–visible spectra of marennine produced by *H. ostrearia* strains from the Pacific and the Mediterranean coasts shows. In the end, the present study confirms that *H. karadagensis* is the species that produces the most different marennine-like pigment*,* and that the pigments produced by all blue *Haslea* species studied so far belong to the same family of organic molecules.

The question that still does not have a clear answer is: Why do the diatoms synthesize blue pigments like marennine? One hypothesis is that producing and excreting marennine is an overflow metabolism, a way to decrease an excess of reducing power inside the cells and to get rid of an excess of carbon fixed by photosynthesis, so it could be a sort of “waste” that needs to be discarded, as most of its mass is constituted by sugars. But then why the blue color? Is it also a sort of sunscreen that protects from excessive luminosity? Is it a radical scavenger, as the antioxidant activity may indicate? Does it come from horizontal gene transfers of bacterial DNA—for instance, from *Pseudomonas aeruginosa* that produces pyocyanin—in ancestral times? On the other hand, considering the antiviral and antibacterial activities, marennine-like pigments could have a clear allelopathic role of defense against viruses, bacteria, and other competitor microalgae, which could reveal a competitive advantage when released in the sea. To be soluble in water, both inside and outside the cell, if the blue chromophore is an organic molecule, it should be surrounded by hydrophilic oligosaccharides (or proteins, but the protein nature was excluded a long ago and confirmed by recent studies).

Given the chemical complexity of the envelope surrounding the chromophore, it has not been identified yet despite ongoing research efforts, which focus on investigating alternative purification techniques. What is currently available are the spectroscopic signatures, which are not significantly different between strains and species, to the exception of *H. karadagensis*.

## 3. Materials and Methods

### 3.1. Blue Haslea Strain Origin and Cultivation

All strains used in this study were clonal cultures derived from single cells isolated using an inverted microscope and glass micropipettes from natural samples of biofilms developed on immersed surfaces (e.g., seaweeds, sediments) collected in marine environments. Geographically different strains of the marennine-producing species *Haslea ostrearia* were sampled in the Bay of Bourgneuf in the Atlantic coast of France (BB), in the San Diego Bay on the Pacific coast of California (SD), and in Puerto de Mogán in Gran Canaria (Canary Islands, Spain) (PM). Regarding the different marennine-like pigment-producing species, *Haslea provincialis* was collected in the Bay of Calvi (Corsica, France), *Haslea silbo* in the Morehead City Channel in North Carolina (USA), *Haslea karadagensis* was isolated in the Black Sea (Crimea, Ukraine)*,* and the not yet characterized *Haslea* sp. nov. was sampled in Puerto de los Abrigos in Tenerife (Canary Islands). Species identification was conducted using molecular barcoding (*rbc*L sequences) and morphological characterization (scanning electron microscopy analyses of frustule striation density), as previously described [3,4,6].

After collection and isolation, all *Haslea* strains and species were cultured at Le Mans University (Le Mans, France) in sterilized, artificial seawater made from a commercial sea salt blend (Instant Ocean, Blacksburg, VA, USA, Aquarium Systems^®^). This solution had a pH of 7.6 ± 0.2 and a salinity of 32 ppm, as proposed by the method of Falaise et al. [36], with an enrichment solution as described in [37]. Cultures were conducted in 500 mL Erlenmeyer flasks, each containing 250 mL of medium and maintained at 16 ± 1 °C in a temperature-controlled chamber. Growth irradiance was 100 µmol m^−2^ s^−1^, supplied by Philips TLD 36 W/965 fluorescent lights, following a 14 h/10 h light/dark cycle.

### 3.2. Blue Haslea Pigment Production, Extraction, and Purification

The different supernatants were cleared of cellular debris using Grosseron^®^ paper filters having 15 µm and 1.4 µm cut-offs (Coueron, France). Subsequently, a distinctive precipitation process using an acid and a base was undertaken, as defined in the patent FR2019/052933, with the final concentration of the filtered supernatant. The obtained blue aqueous concentrate was then dialyzed against ultrapure water for 24 h (MWCO 1 kDa, Spectra/Por^®^). Retentate purification was carried out using a 20 g C18 solid-phase extraction (SPE) (Fisher Scientific^®^, Illkirch, France). The SPE procedure comprises several steps: Initially, the column was activated by eluting a defined volume of methanol, thereby facilitating interactions between the adsorbent and the stationary phase. Subsequently, the conditioning of the stationary phase was carried out with a volume of water acidified with 0.5% trifluoroacetic acid (TFA), preparing the column for the adsorption of *Haslea* pigment. In the following step, the pigment solution was adsorbed through the column, followed by a washing step with water containing 0.5% TFA to remove weakly retained polar compounds from the stationary phase while retaining the bound blue pigment. This was then eluted with a 50% ethanol (0.5% TFA) 50% water mixture, until the solution became colorless. The eluate was then dried using the Hei-Vap rotary evaporator HEIDOLPH^®^ to obtain pure blue *Haslea* pigment in powder form.

### 3.3. UV–Visible Spectrometry

The studies were conducted using an xMark™ absorbance spectrophotometer on 96-well microplates, over a wavelength range of 350–750 nm. A potassium phosphate buffer solution (0.2 M) with a pH range of 1 to 10 was used to investigate the impact of pH on absorption spectra. Disodium phosphate (Na_2_HPO_4_) and monosodium phosphate (NaH_2_PO_4_) were mixed to prepare buffers. The pH was adjusted where necessary with solutions containing 1 M HCl or 1 M NaOH.

### 3.4. Cyclic Voltammetry

Electrochemical experimentation on the *Haslea* strain referenced earlier used a glassy carbon working electrode, a counter electrode made of platinum wire, and an Ag/AgCl reference electrode. The experiments were carried out in 0.1 M phosphate buffer solutions. The pH levels of these solutions were carefully modified to be between 1 and 4 by introducing HCl, and to maintain uniform ionic strength, 0.1 M KCl was added to the solutions.

### 3.5. Infrared Spectrometry

The Fourier-transform infrared (FT-IR) spectra were obtained using a Bruker^®^ (Billerica, MA, USA) Vertex 70v vacuum spectrometer equipped with a DTGS (Deuterated alanine-doped Tri-Glycine Sulphate) detector. Approximately 10 mg of dry marennine and marennine-like pigments were mixed with dry potassium bromide (KBr, 0.2 mg) and processed in a press. The resulting pellets were analyzed. Data collection was performed in transmission over the wavenumber range 400–4000 cm^−1^, averaging 20 scans per spectrum with a spectral resolution of 2 cm^−1^. The acquisitions were carried out using Opus Version 7.5 software.

### 3.6. Resonance Raman Spectroscopy

The Raman measurements were performed at room temperature under a microscope, in backscattering geometry, using a WITec Alpha 300 R confocal Raman spectrometer (WITec GmbH, Ulm, Germany). To prepare the samples, capillaries were preliminary immersed for 2 s in a solution of marennine or marennine-like pigments then removed to place them horizontally on a glass slide to air dry, following the methodology described in Francezon et al. [19]. During the Raman analysis, a Zeiss (Oberkochen, Germany) EC Epiplan-Neofluar^®^ 50× objective (numerical aperture of 0.55) was used to focus the 532 nm line of a Solid-State Sapphire laser (Coherent Inc., Santa Clara, CA, USA) onto the pigments. After verification that no particular modification of the Raman signals occurred for integration times of 2 to 10 min, the laser power was set up to 0.2 mW and the Raman spectrum was collected four times on the same area with an integration time of 120 s. The spectral analysis was performed using WITec Project FIVE plus software (version 5.248, WITec GmbH, Germany) and the Raman spectra are presented, without any baseline correction, in the wavenumber range 300–1800 cm^−1^.

### 3.7. Nuclear Magnetic Resonance

Nuclear magnetic resonance (NMR) spectra were recorded on a Bruker Avance III 400 MHz (9.4 T) spectrometer equipped with a 5 mm BBFO^+^ probe (^13^C DEPT, ^1^H spectra of *H. silbo* and *H. sp. nova*) and a Bruker Avance Neo 500 MHz (11.7 T) spectrometer equipped with a 5 mm SmartProbe BBFO probe (all other ^1^H spectra). Between 2 mg and 5 mg of sample material were dissolved in 500 µL D_2_O for the proton 1D spectra. A total of 128 scans were acquired with presaturation. For the ^13^C DEPT spectrum the delays were set according to an average *J* of 145 Hz. 80 K scans were accumulated with a total interscan delay of 3.1 s.

### 3.8. Energy-Dispersive X-Ray Spectroscopy

*Haslea* blue pigment powders were analyzed by energy-dispersive X-ray spectroscopy (EDX) and scanning electron microscopy (SEM). The SEM was equipped with an Oxford model energy-dispersive X-ray spectrometer (EDS), which uses an electron beam with energies ranging from 0.5 to 30 kV. Using Aztec EDX analytical system (Oxford instruments), the elemental atomic composition was analyzed semi-quantitatively and the powders were qualitatively mapped.

## 4. Conclusions

The efforts made in bioprospection in recent years have led to the discovery of new blue *Haslea* species and strains. In this research work, the spectral characterization of the pigments produced by different strains of blue *Haslea*, encompassing three strains of *H. ostrearia* with different geographical origin (BB, SD, and PM); *H. karadagensis*, *H. provincialis*, and *H. silbo*; and a species not characterized yet, *Haslea* sp. nov. was carried out. Complementary techniques such as nuclear magnetic resonance and UV–visible spectrometry were used to investigate the marennine-like pigments and compare them to the best-known marennine from *H. ostearia* (BB). The data showed that the pigments are quite similar in terms of sugar composition of the polysaccharide skeleton, and absorption in the UV–visible, infrared, and Raman region. Cyclic voltammetry responses generated by the chromophore suggest the existence of isomers that have a slight difference but belong to the same family of organic molecules. The most relevant differences were observed for *H. karadagensis*, which so far has only been found in the Black Sea. It would be interesting to continue the investigation of this species as soon as it will be easily available.

## Figures and Tables

**Table 1:** Common band assignments for the FT-IR spectrum of marennine-like pigments from different blue *Haslea* strains and species.

Wave Number (cm^−1^)	Assignment	Reference
3549	Hydroxyl (O-H) stretching	[32]
2850	Aliphatic C-H stretching	[32]
2553	Sulfur-bonded compounds	[33]
1749	Carbonyl (C=O) groups	[32]
1469	C-H bending	[34]
1299	O-C-O asymmetric stretching	[34]
1259	Sulfated carbohydrates	[33]
1100	C-O stretching	[30]

**Table 2:** Summary of vibrational wavenumbers (cm^−1^) observed by Raman spectroscopy of *Haslea ostrearia* (BB), *Haslea provincialis, Haslea ostrearia* (PM), and *Haslea* sp. nov.

*Haslea ostrearia* (BB)	*Haslea provincialis*	*Haslea ostrearia* (PM)	*Haslea* sp. nov.
1462	1461	1463	1462
1526	1534	1527	1527
1569	1569	1569	1569
1604	1594	1604	1602
1650	1650	1650	1650

**Table 3:** Composition of the polysaccharide of *H. provincialis* hydrolyzed pigment compared to that of *H. ostrearia* [17]. Uncertainty estimated to 3 percentage points. Difference to 100%: non-assigned signals.

Type	Fraction *H. provincialis* [%]	Fraction *H. ostrearia* [%] [17]
Rha*p*	38	20
Gal*p*	22	36
Xyl*p*	20	19
Man*p*	11	8
Fuc*p*	8	8

## Data Availability

All data analyzed during this study are included in this article and in the Supplementary Information.

## Supplementary Materials

The following supporting information can be downloaded at https://data.mendeley.com/datasets/ky2bdpskpz/4 (published: 17 October 2024).
